# A Review of Genomic Testing and SDH‐ Deficiency in Gastrointestinal Stromal Tumors: Getting to the GIST

**DOI:** 10.1002/cam4.70669

**Published:** 2025-02-10

**Authors:** Vaia Florou, Michelle F. Jacobs, Ruth Casey, Denisse Evans, Becky Owens, Margarita Raygada, Sara Rothschild, Samantha E. Greenberg

**Affiliations:** ^1^ Division of Oncology, Department of Internal Medicine, Huntsman Cancer Institute University of Utah Salt Lake City Utah USA; ^2^ Division of Genetic Medicine, Department of Internal Medicine University of Michigan Ann Arbor Michigan USA; ^3^ Department of Medical Genetics Cambridge University Cambridge UK; ^4^ Life Raft Group Wayne New Jersey USA; ^5^ Pediatric Oncology and Neuro‐Oncology Branch National Cancer Institute/National Institutes of Health Bethesda Maryland USA; ^6^ Department of Health Care Sciences UT Southwestern Medical Center Dallas Texas USA

**Keywords:** gastrointestinal stromal tumor (GIST), genomic testing, hereditary paraganglioma‐pheochromocytoma, SDH‐deficient GIST, succinate dehydrogenase complex

## Abstract

Gastrointestinal Stromal Tumors (GISTs) have seen significant advancements in their diagnosis and management, driven by targeted therapeutic development and molecular testing. The identification of mutations in genes such as KIT and PDGFRA has transformed treatment approaches, particularly through targeted therapies like imatinib, which have improved patient outcomes. This review explores the critical role of genomic testing in GIST, highlighting its importance in accurate diagnosis, treatment planning, and long‐term surveillance for KIT/PDGFRA negative, SDH‐deficient GISTs. SDH‐deficient GISTs arise from mutations or epigenetic changes affecting the succinate dehydrogenase complex. The complexity of SDH‐deficient GISTs, including their association with hereditary syndromes such as Hereditary Paraganglioma‐Pheochromocytoma and/or hypermethylation of the *SDHC* promoter, underscores the need for comprehensive germline testing. Despite the availability of guidelines, variability exists in genomic testing recommendations across different regions, necessitating a unified approach. This review proposes a simplified algorithm for the genomic workup of GIST, and suggests all individuals with SDH‐deficient GIST, regardless of germline testing result, require monitoring for additional *SDHx*‐related tumors, given the lack of widely available methylation and full gene *SDHA* analysis.

## 1 Introduction

Over the past decade, the understanding and management of Gastrointestinal Stromal Tumors (GIST) has undergone significant evolution. Advances in genomic research have reshaped the landscape of GIST diagnosis and treatment as our knowledge of the molecular underpinnings of GIST has expanded. Similarly, we can now target these tumors precisely, improving patient outcomes and offering new hope for those affected by GIST.

Genomic molecular testing is a critical component of GIST workup and treatment. The identification of specific genetic mutations, such as those in the KIT and PDGFRA genes, has been instrumental in guiding the use of targeted therapies like imatinib. Molecular profiling is crucial for the accurate diagnosis of GIST and for determining treatment regimens and long‐term surveillance protocols. As such, genomic testing has become an indispensable tool in the oncologist's arsenal to tailor patient care.

Here, we review the role of genomic testing in the management of GIST. We will explore the latest advancements and discuss their practical applications in clinical settings. Furthermore, given shifts in access and affordability of testing, we propose multiple mechanisms for the utilization of germline testing in clinical care. Simultaneously, there are limitations to genomic testing that must be acknowledged by the care team. By taking advantage of the current genomic landscape of GIST, we can ultimately improve outcomes for patients with GIST and their families.

## 2 Overview of GIST

Gastrointestinal (GI) stromal tumor (GIST) is a sarcoma of the gastrointestinal tract in adults, affecting approximately 4000–6000 new patients annually in the United States [1], with an incidence of approximately 6–22 cases per million per year. GISTs originate from the interstitial cells of Cajal [2] and develop along the GI tract. They predominantly harbor gain‐of‐function mutations in the *KIT* or *PDFGRA* genes [3, 4], leading to constitutive activation of the KIT or PDGFRA receptor tyrosine kinases, respectively. Approximately 10%–15% of GISTs are driven by alternative mechanisms, such as inactivating mutations in the *NF1* gene [5] or genes encoding the succinate dehydrogenase (SDH) subunits, collectively referred to as wild‐type GISTs [6]. Further delineating wild‐type GISTs by showing a loss of succinate hydrogenase and SDHB immunohistochemistry (IHC) establishes whether the GIST is SDH‐deficient.

Most GISTs are diagnosed in adulthood, with fewer cases occurring in children or young adults. Early‐onset GIST often occur due to germline pathogenic variants (mutations) in the *SDH* genes [7] or promoter methylation of the *SDHC* gene [8].

GISTs are typically well‐circumscribed tumors that arise from the muscularis propria. About 70% of GISTs comprise spindle cells, 20% of epithelioid cells, and the remaining show mixed morphology [9]. On IHC for diagnostic purposes, KIT is expressed in 95% of the cases, DOG‐1 in 98%, PDGFRA in 80%, and CD34 in 70%–80% of GISTs [9]. When SDHB IHC is absent, a tumor is considered SDH‐deficient GIST [10]. To determine the type of GIST at time of diagnosis, a combination of IHC and genomic testing is recommended [11].

## 3 Genomic Testing for GIST Tumors

Genomic testing occurs at the time of diagnosis, as GIST treatment differs based on the findings from mutational analysis of the tumor itself. In Table 1, we outline the types of genomic workup conducted in clinical GIST management. Generally, somatic testing, which involves molecular tumor analysis to identify pathogenic variants driving cancer growth, has become standard for mutational analysis in GIST. Somatic testing identifies mutations in the tumor itself, aids in prognostication, predicts treatment response, and informs surveillance strategies (Table 1). Somatic genomic testing involves either targeted panels that focus on specific genes known to drive GIST, such as *KIT* and *PDGFRA*, or broader panels that assess a wider array of genes for a more comprehensive analysis. Broader somatic testing can sometimes reveal secondary findings, such as potential germline mutations in genes like *BRCA2*, necessitating further germline testing for hereditary risks.

**TABLE 1:** Description of genomic testing used in GIST diagnosis and management. The type of tissue used for genomic testing, and its impact on GIST treatment and/or family members of the affected relative, are also discussed. IHC: Immunohistochemistry.

Type of genomic testing	Brief definition	Tumor or normal tissue?	Impact to treatment?	Impact to family?
Somatic	Molecular tumor analysis that identifies pathogenic variants suspected to drive cancer growth	Tumor	Yes	No^a^
KIT/PDGFRA	Next‐generation sequencing of the *KIT* and *PDGFRA* genes	Tumor	Yes	No
SDHB IHC	Antibodies used to detect specific SDHB proteins in the tumor sample	Tumor	Yes	No^a^
Germline	Performed on DNA in healthy cells, identifies hereditary risk that increases a person's risk for additional tumors and/or risk to family members	Normal	Potentially	Yes
Paired tumor/normal	Somatic sequencing tests that include germline testing in their analysis	Both	Yes	Yes

^a^ Germline testing is required as follow‐up to determine the impact to family members.

In addition, IHC detects SDH‐ deficiencies in GIST tumors. The loss of function of any SDH subunit leads to the loss of SDHB expression by IHC, making this a useful diagnostic marker for SDH‐deficient GIST [12]. In cases of SDH‐deficient GISTs or when somatic testing suggests germline variants, confirmatory germline testing on healthy cell (normal) DNA is recommended to assess hereditary cancer risks.

## 4 SDH‐Deficient GIST

An SDH‐deficient GIST, defined by the absent or impaired function of the SDH enzyme, is caused by germline mutations in the *SDHx* genes or epigenetic methylation of the *SDHC* promoter. The SDH enzyme complex comprises four subunits (SDHA, SDHB, SDHC, SDHD), playing a crucial role in converting succinate to fumarate. Mutations in the *SDH* genes lead to the inactivation of SDH and the accumulation of succinate in the mitochondria [13]. Increased succinate acts as an oncometabolite, stabilizing hypoxia‐inducible factor 1 a (HIF1a), which then promotes the transcription of genes involved in angiogenesis, proliferation, and glycolysis [13]. SDH‐deficient GISTs result more commonly from loss‐of‐function mutations in an *SDH* gene (80% of the cases) or less frequently from *SDHC* promoter methylation (approximately 20% of the cases) [14]. No systemic therapy is approved explicitly for SDH‐deficient GIST at the time of this review.

Based on current evidence, all individuals with an SDH‐deficient GIST, regardless of etiology (described below), are at risk for associated tumors such as paragangliomas, renal cell carcinomas, and other *SDHx*‐related tumors, and should be monitored according to established guidelines [11, 15].

## 5 Molecular Etiology of SDH‐Deficient GIST

Most cases of SDH‐deficient GIST are attributed to a germline *SDHx* variant (~80%), and the remainder (~20%) are caused by tumoral hypermethylation of the *SDHC* promoter region [6]. While multiple terms for the underlying conditions associated with SDH‐deficient GIST exist, we will define and standardly use GIST lacking SDHB IHC expression as SDH‐deficient GIST, germline mutations in the *SDHx* genes as a subset of the broader condition Hereditary Paraganglioma‐Pheochromocytoma (HPGL) [16, 17], and epigenetic methylation of the *SDHC* promoter region as epimutant [18]. Here, we describe the current evidence surrounding each etiology of SDH‐deficient GIST and define GIST by mutational status (Table 2).

**TABLE 2:** Defining GIST by mutation status, SDH‐deficient status, and germline testing outcomes.

Type of GIST	Mutation Type Associated with GIST	Also Known As…	Somatic KIT	Somatic PDGFRA	SDH IHC	SDHx Germline	Promoter Methylation	Notes
KIT/PDGFRA Mutant GIST	KIT mutated GIST		+	−	+	−	−	
	PDGFRA mutated GIST		−	+	+	−	−	
SDH‐competent GIST	Wildtype GIST		−	−	+	−	−	Can be related to NF1, BRAF, Fusions, etc
SDH‐deficient GIST	SDHC Promoter Methylation	Carney Triad	−	−	−	‐ (+ **in 10**%)	+	Follow SDHx germline management in patient; no family testing needed
	Hereditary KIT & SDHx		+	−	−	+	−	Only reported in one family
	SDH germline	Hereditary paraganglioma/pheochromocytoma; Carney Stratakis	−	−	−	+	−	Tumor risks vary by SDHx gene

### 5.1 Germline SDHx Variants in GIST

Pathogenic *SDHA* variants (PVs) account for up to 50% of SDH‐deficient GIST cases, and PVs in *SDHB/C/D* account for the remaining 20%–30% cases of hereditary SDH‐deficient GIST [6]. Given SDH‐deficient GIST are associated with PVs in all of the *SDHx* genes (*SDHA/SDHB/SDHC/SDHD*), comprehensive germline testing for patients with an SDH‐deficient GIST via testing of the *SDHx* genes is recommended [11]. Importantly, variable and incomplete penetrance of the *SDHx* genes [19, 20] means that a family history of GIST or paraganglioma is often lacking and should not dissuade from the role of germline testing in an individual with Carney‐Stratakis syndrome or an SDH‐deficient tumor.

Tumor risk due to germline pathogenic variants in the *SDHx* genes are part of a broader condition known as hereditary paraganglioma‐pheochromocytoma syndrome (HPGL). Though a misnomer for SDH‐related GIST, HPGL broadly covers the increased risk for multiple tumors, including paraganglioma, GIST, and renal cell carcinoma. In addition, PVs in *SDHAF2, MAX, FH, VHL, RET, DNMT3A, SLC25A11, MDH2* and *TMEM127* confer increased risk for paraganglioma and pheochromocytoma, the most commonly occurring tumor in people with HPGL [17]. For individuals with HPGL who develop GIST, their GIST may occur at a younger age, have lymph node involvement, grow slowly, be multifocal, and/or more frequently metastasize [11, 17].

Identifying germline PVs in SDH‐deficient GIST affects both treatment and family members who may also have a germline *SDHx* PV. A timely diagnosis of HPGL can impact treatment, as SDH‐deficient GISTs are largely resistant to TKIs, and may have a higher likelihood of response to sunitinib [11]. For those with a germline *SDHx* PV, germline testing of blood relatives can determine additional individuals at increased risk to develop HPGL‐related tumors. This familial germline testing, also known as cascade testing, can be completed through genetic counseling. For family members who have a germline *SDHx* PV consistent with HPGL, increased screening due to their higher risk of tumor development is recommended by multiple consensus guidelines [15, 21]. Notably, individuals with maternally inherited *SDHD* PVs should undergo cascade testing, but do not require screening due to predominantly parent‐of‐origin effects [22]. Family members who do not have the germline PV are at general population risk for tumor development and are not recommended to undergo high‐risk screening.

### 5.2 SDH Promoter Methylation in GIST

Carney's triad refers to a non‐hereditary tumor syndrome consisting of pulmonary chondroma tumor, paraganglioma and SDH‐deficient GIST [7, 23]. Initially, a large somatic genomic deletion on 1q, encompassing the *SDHC* gene locus, was postulated as the molecular driver of this tumor syndrome [8]. However, later research [24] confirmed that an epimutation of the *SDHC* gene due to hypermethylation of the promoter region, rather than a sequence mutation or large deletion, was implicated in Carney's triad [24]. While the initial diagnosis of Carney Triad arose from individuals with all three tumors, a Carney Triad diagnosis requires one of three things: (1) chondroma plus GIST in same patient; (2) chondroma plus PGL in same patient; (w3) confirmed *SDHC* promoter epimutation (currently not available commercially) [25]. *SDHC* epimutations are more commonly implicated in SDH‐deficient GIST compared to SDH‐deficient paraganglioma/pheochromocytoma and are almost exclusively identified in female patients [26]. In two large studies, SDHC epimutations accounted for 20% of SDH‐deficient GIST [6, 26]. This may be an underestimation, given limited access to SDHB IHC and *SDHC* promoter methylation analysis.

Notably, 10% of patients with germline variants in the *SDHx* genes have also manifested the clinical phenotype of Carney's triad. Studies have confirmed that *SDHC* epimutations can occur in conjunction with a co‐existing pathogenic germline variant in one of the *SDHx* genes [26]. This is an important clinical observation and indicates that germline testing is necessary even with a clinical phenotype of Carney's triad. *SDHC* hypermethylation is not believed to be constitutional or hereditary, although there is limited evidence in this regard [26]. At present, commercial screening for an *SDHC* epimutation is not available.

### 5.3 Other Causes of SDH Deficient GIST

SDH‐deficient GISTs can also arise from other genetic alterations affecting the succinate dehydrogenase (SDH) complex. Somatic SDH variants in the tumor tissue can lead to loss of SDH function [27]. Similarly, mosaicism, where the mutation occurs in some but not all cells of the body, can also cause SDH deficiency on IHC [24, 26].

## 6 Current Guidelines for the Genomic Workup of SDH‐Deficient GIST

Despite the importance of genomic testing in managing GIST and its potential germline implications, there is significant variability in the recommendations for somatic and germline genomic testing for SDH‐deficient GIST across different guidelines. A summary of current guidelines is described in Table 3, and the specific recommendations from each guideline are included below.

**TABLE 3:** Summary of existing guidelines for recommendations of SDHB immunohistochemistry (IHC) and molecular tumor analysis, and timing in relation to KIT/PDGFRA testing.

Country	Organization	Year	During Diagnostic Process		After Negative KIT/PDGFRA Testing		Germline Testing	Genetic Counseling
			SDHB IHC	Molecular Tumor Analysis	SDHB IHC	Molecular Tumor Analysis	Multi‐gene panel	
United States	NCCN	2024	X	X	X	X	X	X
Spain	GEIS	2023	X	X		X		
UK	British Sarcoma Group	2024		X	X		X	
Japan	Japan Society of Clinical Oncology	2022			X	X		
Europe	ESMO & Partners	2021		X	X			
Europe & Latin America	SELNET	2021						
France	French InterGroup	2019		X	X		X	X

Abbreviations: ESMO, European Society of Medical Oncology; GEIS, Spanish Group for Sarcoma Research; NCCN, National Comprehensive Cancer Network; SELNET, Sarcoma European Latin‐American Network.

The United States‐based National Comprehensive Cancer Network (NCCN) recommends SDHB IHC and mutational analysis of all GISTs [11]. For SDH‐deficient GISTs and GISTs with somatic *SDH* mutations on broad molecular tumor analysis [11], a referral to a genetic counselor for germline testing and assessment is recommended. The Spanish Group for Sarcoma Research (GEIS) recommends mutational analysis of all GISTs, with the French Intergroup Clinical Practice recommending subsequent SDHB IHC testing if a *KIT* or *PDGFRA* mutation is not detected [28, 29]. Further genetic consultation is recommended if there is loss of SDHB expression, a family history of GIST, diagnosis of multiple GISTs, age of diagnosis less than 30 years, or association with paraganglioma [29]. Like other European guidelines, the clinical practice guidelines of the European Society of Medical Oncology (ESMO) recommend SDHB IHC in GISTs without *KIT/PDGFRA* mutations [30]. The British Sarcoma Group [31] also recommends a minimum of KIT/PDGFRA molecular analysis in the diagnostic workup of all GIST, with SDHB IHC in all patients with GIST harboring features suggestive of SDH deficiency. If SDHB IHC is absent, guidelines recommend testing for sporadic or germline SDH mutation/epigenetic loss [31]. Sarcoma European Latin‐American Network (SELNET) guidelines for GIST recommend *KIT* and *PDGFRA* tumor mutational analysis for candidates for systemic therapy [32] but does not provide specific guidelines on SDH IHC testing. Lastly, the Japanese Clinical Practice Guidelines for GISTs [33] recommend upfront SDHB IHC testing in all epithelioid subtype GISTs and/or when *SDH* gene abnormality is suspected. Somatic gene analysis is recommended in KIT‐negative or weak KIT‐positive GISTs and primary imatinib‐resistant GIST [33].

**FIGURE 1:**
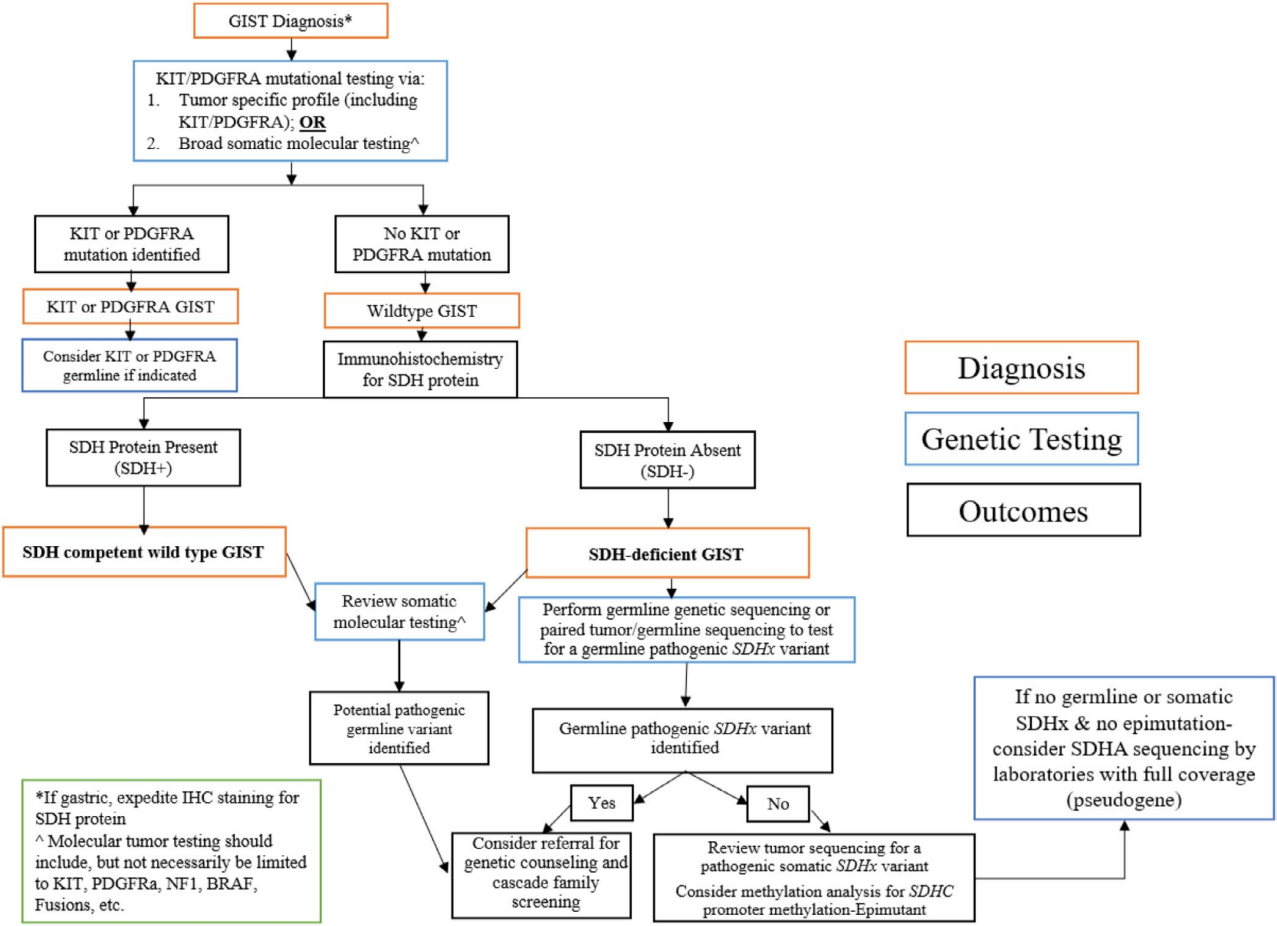
Author‐recommended process to evaluate GIST via mutational testing and/or SDH workup with subsequent germline testing needs. IHC, Immunohistochemistry.

Given the complexity and heterogeneity in genomic testing guidelines for SDH‐deficient GIST, we propose a simplified, consolidated algorithm (Figure 1). Upon histopathologic diagnosis of GIST, we recommend either targeted *KIT/PDGFRA* gene testing or broad somatic molecular testing. In the absence of a *KIT/PDGFRA* mutation, SDHB IHC should be performed next. If SDH‐deficient GIST is confirmed, germline genetic sequencing is recommended.

## 7 Additional Considerations for Germline Testing for GIST Tumors

In contrast to SDH‐deficient GISTs, most KIT/PDGFRA‐mutated GIST diagnoses are sporadic. However, a few GIST diagnoses are associated with hereditary conditions. Clues to hereditary risk for KIT or PDGFRA‐mutated GIST include multiple GISTs in the same person or in multiple family members, and/or young age of diagnosis. The finding of the same somatic mutations in multiple primary tumors is also suspicious for hereditary risk. Beyond HPGL, hereditary conditions that confer an increased risk of developing GIST include:

- Germline *KIT* PV: Hyperpigmentation has been described in families with germline pathogenic variants in *KIT* presenting with GIST [34]. Germline pathogenic variants in *KIT* have only been described in a small number of families with GIST, as well as some families with mastocytosis [35]. Some genotype–phenotype correlations have been suggested, but no definitive relationships have been determined due to the rarity of this finding [35].
- Germline *PDGFRA* PV: Germline pathogenic variants in *PDGRFA* are extremely rare. A case series of five families describes affected individuals with coarse facies and skin, broad hands and feet, and premature tooth loss [36]. Affected individuals had multiple GISTs, although this condition appears to exhibit incomplete penetrance [36].
- Germline *NF1* PV—Neurofibromatosis type 1 (NF1): Clinical findings of this condition include café au lait macules, cutaneous neurofibromas, Lisch nodules, choroidal abnormalities, intertriginous freckling, learning difficulties, with penetrance of this condition being close to 100% [37]. GIST occurs in approximately 7% of individuals with NF1, and typically presents as multiple GISTs in small intestine [38, 39].

In addition to non‐SDH germline testing considerations, the detection of variants in the genuine *SDHA* gene can be complicated by the presence of several pseudogenes (e.g., *SDHAP1*, *SDHAP2*, *SDHAP3*) [40]. Due to this complexity, commercial genetic testing laboratories typically offer *SDHA* sequencing but lack appropriate assays to evaluate for deletions or duplications of the *SDHA* gene. Despite clear statements in their reports, many patients are incorrectly informed that their germline panel is negative, and no further follow‐up is needed. For those without identified germline sequencing variants, particularly those with an *SDHA* mutation on mutational tumor testing, *SDHA* full gene analysis should be conducted. Full gene analysis must include deletion and duplication analysis and be conducted by a laboratory that offers this comprehensive service. Consideration of *SDHA* dosage analysis should be made with a genetic specialist to facilitate this process.

## 8 Service Delivery Options for Delivery of Genomic Services

While multiple guidelines recommend a minimum of KIT/PDGFRA testing, others endorse broader molecular mutational analysis. Similarly, once a suspicion of hereditary tumor risk occurs, genetic education is recommended prior to germline testing. However, the use of genetic services into clinical GIST management varies widely. While somatic testing is primarily facilitated by the oncology care team, germline testing can be completed through a genetic specialist or the oncology care team. Additionally, patient education can occur before testing to introduce possible testing outcomes, or after testing, summarizing testing results. Below, we discuss service delivery options for GIST genomic testing.

### 8.1 Pre‐Test Counseling

Traditionally, oncology providers refer individuals with *SDH‐*deficient GISTs to a genetic counselor for a pre‐test counseling session, facilitation of germline testing, and post‐test result disclosure. However, the addition of a care team member, or the extra step of pre‐test counseling before testing, can reduce uptake of testing. All individuals are recommended to pursue germline testing with *SDH*‐deficient GISTs. Therefore, it is imperative to maximize germline testing uptake and we must consider alternative options for germline testing service delivery.

### 8.2 Point‐Of‐Care Germline Testing

Many institutions now include germline testing during the oncology visit, which may include provider discussion with the patient, video education, and/or chatbot mechanisms [41, 42]. When a patient meets criteria, the oncology provider discusses and facilitates testing, and collaborates with the genetic specialist for result disclosure. This model reduces the additional steps in the pre‐test process, thereby improving uptake of germline testing amongst patients [43]. A point‐of‐care approach to germline testing requires provider education, collaboration with an oncology care team member to facilitate germline testing, and consultation with a genetic specialist for complex test results. Each team should analyze their available resources, rate of germline testing uptake, patient care processes, and other institutional factors to decide the best mechanism for them.

### 8.3 Somatic Testing

Somatic testing is primarily initiated amongst the oncology care team. This may be done sequentially, occurring before germline testing, or simultaneously if KIT/PDGFRA testing is negative. A high variant allele frequency in a gene that indicates a potential germline variant should result in collaboration with genetics to facilitate the optimal testing [44]. In general, tumor‐detected pathogenic variants cannot be used to infer germline origin and negative tumor sequencing does not imply a negative germline result. Pathogenic germline PVs may go unreported in somatic panels due to disparities in pathogenicity definitions between somatic and germline laboratories, gene coverage limitations in the somatic assay design, and variations in bioinformatic filtering [45].

### 8.4 Suspicion of Germline PV From Somatic Testing

Germline testing is recommended for *SDHA, SDHB, SDHC*, and *SDHD* across all ages and tumor types in the setting of SDH‐deficient staining and/or when an *SDHx* PV is identified in the tumor [11, 30]. Additionally, the NCCN guidelines recommend follow‐up germline testing if a pathogenic/likely pathogenic (P/LP) variant found in the tumor has clinical implications for a matching germline PV [46]. Labs vary in how they report potential germline findings through paired tumor/normal testing or recommend confirmation for incidentally discovered variants. The extent of germline analysis varies in paired‐normal testing and may require additional testing to fully evaluate hereditary cancer syndromes. It is crucial to complete germline testing through a lab optimized for such analyses to ensure accurate interpretation and appropriate follow‐up.

## 9 Challenges in Diagnostic and Genomic Testing for GIST

Genetic testing‐both germline and somatic‐ is essential for clinicians to make informed decisions regarding surgery, preventive screening, and treatment. However, as the landscape of genetic testing for GIST has evolved, new challenges have emerged, requiring additional strategies to optimize clinical care. Below, we discuss these challenges:

### 9.1 Importance of Timely and Comprehensive Diagnostic Workup

Delays in comprehensive diagnostic workup for GIST create challenges for effective patient management and optimal treatment outcomes. Early and accurate identification of specific tumor mutations, such as KIT, PDGFRA, or SDH deficiencies, allows for tailored therapeutic strategies that can significantly improve prognosis and survival rates [47, 48, 49, 50]. Delayed or incomplete diagnostic workup can lead to suboptimal treatment choices, prolonged symptoms, and delays for both the patient and their family members in terms of additional surveillance. Therefore, comprehensive and prompt GIST diagnostic workups are essential. SDH‐deficient GIST diagnoses are often delayed, as this subtype goes unrecognized due to limited knowledge amongst healthcare providers. Many patients do not continue testing after receiving a negative result for KIT or PDGFRA mutations, leading to delays in diagnosis and treatment [51].

The SDH‐deficient GIST diagnostic algorithm, developed by the Life Raft Group (LRG) and used in the College of American Pathology (CAP) and NCCN guidelines, demonstrated that implementing SDHB IHC staining for all gastric tumors significantly increased the diagnosis rate of SDH‐deficient GIST by 60% [52]. Therefore, it is essential to enhance education and awareness amongst healthcare providers.

### 9.2 Discrepancies Between Consensus Guidelines for Genomic Testing in GIST

Discrepancies amongst international guidelines for genetic testing of GIST patients also present a challenge in navigating guidelines. There is a strong need for consistent international guidelines generated from experts across the world, and the author group strongly recommends an international consensus panel be convened for standardization of guidelines.

### 9.3 Heterogeneity in Phenotype and Variant Interpretation

Understanding the complexities of genetic variations and their clinical implications is crucial for decision‐making and patient education. This section delves into the intricacies of phenotypic heterogeneity, incomplete penetrance, and the management of variants of uncertain significance (VUSs) in SDH‐deficient GISTs. By examining these aspects, we can better appreciate the challenges and considerations necessary for accurate diagnosis and patient care.

#### 9.3.1 Phenotypic Heterogeneity and Incomplete Penetrance

The clinical spectrum of SDH‐deficient GIST ranges from indolent to rapidly advancing. At the same time, age‐related penetrance for HPGL‐related tumors varies significantly by gene [25, 27]. Whereas individuals with paternally‐inherited *SDHD* PVs have an SDH‐related tumor risk over 40% [20], the risk for SDH‐related tumors in someone with an *SDHA* PV may be as low as 1.7% [19]. Given that *SDHA*‐related GIST is the most common hereditary diagnosis from germline GIST workup [53], individuals may or may not have other relatives with SDH‐related tumors, challenging the utility of family history in decision‐making. This variability also leads to misinterpretation of the genetic etiology of these tumors, which is further complicated by the pattern‐of origin effect in the *SDHD* gene. Due to these factors, many SDH‐deficient GIST patients with *SDHx* PVs have a negative family history and do not undergo germline testing. Though diagnosed with ‘sporadic GIST’, patients with SDH‐deficient GIST and negative germline results often have hypermethylation of the SDHC promoter region or a germline *SDHA* PV not detected in multigene panels. Regardless, an SDH‐deficient GIST diagnosis indicates an increased risk for SDH‐associated tumors and requires additional monitoring.

#### 9.3.2 Managing Variants of Uncertain Significance (VUSs) in GIST Genes

VUSs represent a significant proportion of reported germline variants in genomic studies and databases like ClinVar [54, 55]. Currently, most reclassified VUSs are found to be benign, with a small percentage being upgraded to pathogenic [55, 56]. Current guidelines for genetic practitioners advise that clinical decisions should not be based on VUSs [57]. However, VUSs in the SDH genes should be interpreted with caution as several VUSs in this population co‐segregate with the disease (unpublished data). Genetic counseling for VUSs in the *SDHx* genes is recommended. Functional testing including the use of SDHB IHC and tumor and serum metabolomics may aid the interpretation of VUSs in *SDHx* genes in the future [58, 59, 60].

## 10 Conclusion

Given the complexities of genomic testing for GIST and the need for timely diagnosis of SDH‐deficiency, increasing awareness of the need for comprehensive genomic GIST workup are imperative. The heterogeneity and variable etiology of GISTs often lead to misdiagnoses, misconceptions, and inappropriate treatment strategies. By expanding genomic testing at the time of diagnosis, clinicians can make more informed decisions, identify at‐risk patients, and provide tailored management plans. Even in the setting of negative germline testing, an SDH‐deficient GIST may be due to Carney Triad or incomplete germline testing, warranting long‐term surveillance for other HPGL‐related tumors. Overall, comprehensive genomic analysis is crucial. This approach not only enhances diagnostic accuracy but also optimizes patient outcomes by enabling precise and timely therapeutic interventions for the patient and their family.

## Author Contributions

**Vaia Florou:** conceptualization (equal), writing – original draft (equal), writing – review and editing (equal). **Michelle F. Jacobs:** conceptualization (equal), writing – original draft (equal), writing – review and editing (equal). **Ruth Casey:** conceptualization (equal), writing – original draft (equal), writing – review and editing (equal). **Denisse Evans:** conceptualization (equal), writing – original draft (equal), writing – review and editing (equal). **Becky Owens:** conceptualization (equal), writing – original draft (equal), writing – review and editing (equal). **Margarita Raygada:** conceptualization (equal), writing – original draft (equal), writing – review and editing (equal). **Sara Rothschild:** conceptualization (equal), writing – original draft (equal), writing – review and editing (equal). **Samantha E. Greenberg:** conceptualization (equal), project administration (equal), supervision (equal), writing – original draft (equal), writing – review and editing (equal).

## Ethics Statement

This review did not require human subject research and used publicly available data.

## Conflicts of Interest

Dr. Casey is an editor for the journal Clinical Endocrinology and Endocrine Oncology. The other authors declare no conflicts of interest.

## Précis

Genomic testing in gastrointestinal stromal tumors (GIST) is required and multi‐faceted to ensure comprehensive workup for therapeutic decision‐making. Individuals with SDH‐deficient GIST require additional long‐term follow‐up.

## Data Availability

The authors have nothing to report.
